# Chloride administration in the intensive care unit, an independent predictor of mortality

**DOI:** 10.1186/2197-425X-3-S1-A975

**Published:** 2015-10-01

**Authors:** S Smolders, RJ Bosman, H Endeman

**Affiliations:** Onze Lieve Vrouwe Gasthuis, Pediatric Intensive Care Unit, Amsterdam, Netherlands; Onze Lieve Vrouwe Gasthuis, Intensive Care Unit, Amsterdam, Netherlands

## Introduction

Chloride is the body's most important extracellular anion, important in many physiologic processes including acid base balance and osmotic pressure [1]. Hyperchloremic metabolic acidosis is a common finding in critically ill patients and may be associated with renal failure and even increased mortality [2].

## Objectives

The objective of our study was to determine if chloride administration in a critically ill population is associated with hospital mortality.

## Methods

We performed a retrospective observational study in a 24 bed tertiary mixed medical surgical ICU in a teaching hospital in the Netherlands. Patients admitted to the ICU in the period between January 1th 2008 and November 1th 2014 were screened for eligibility. Inclusion criteria were: 1. ≥ 18 years. 2. Length of stay (LOS) of ≥ 72 hours. Readmissions to the ICU were excluded. The primary end points for our analysis was hospital mortality. Univariate analysis was performed with Wilcoxon rank sum test for nonparametric data. Multivariate analysis was performed with predictors of ICU and hospital mortality (age, Acute Physiology and Chronic Health Evaluation IV predicted mortality) as well as factors associated with (hyper)chloremia, metabolic acidosis and fluid resuscitation. Chloride administration was defined as the total amount of chloride in mmol/l administered to a patient in a given time period.

## Results

We studied 1471 patients. Mean age at admission was 67 years, APACHE II score 24, the average stay 10,5 days (table 1, baseline characteristics). In univariate analysis chloride levels at 72 hours were predictors of hospital mortality as were pH, chloride administration and total fluid administration (table 2). A multivariate analysis was performed to determine whether chloride, chloride administration, fluid administration and pH are independent predictors of mortality. This models shows that chloride levels are not an independent predictor of hospital mortality, chloride administration however is an independent predictor (OR 1.005; 95% CI 1.003-1.007 at 72 hours) as is pH, total fluid administration and age (table 3).

**Table 1:** Baseline characteristics.

	Whole population Mean	Hospital survivors Mean	Non survivors Mean
Age (years)	67 ± 12	67 ± 13	69 ± 12
Sex (male)	64,9%	65,7%	62,6%
APACHE II Score	24 ± 7	22 ± 7	28 ± 7
Admission type medical	59%	53,1%	74,3%
Admission type surgical	41%	46,9%	25.7%
Length of stay at ICU (hours)	254	224	329
Acute renal failure	22.9%	20,4%	29,3%
CPR before admission	12.3%	9,9%	18,6%
Renal replacement therapy during ICU stay	30.6%	25,9%	42,8%

**Table 2:** Univariate analysis

	Hospital survivors Mean	Non-survivors Mean	P
Chloride at admission (mmol/l)	106,6 ± 6,3	106,8 ± 6,5	0,986
Chloride at 72 hours (mmol/l)	106,3 ± 5,1	107,6 ± 5,1	0,000
pH at admission	7,33 ± 0,09	7,30 ± 0,10	0.000
pH at 72 hours	7,44 ± 0,06	7,41 ± 0,07	0.000
Total fluid administered at 24 hours (liters)	4,04 ± 2,6	4,41 ± 2,8	0.027
Total fluid administered at 72 hours (liters)	6,27 ± 3,5	7,58 ± 4,1	0.000
Chloride administered at 24 hours (mmol)	507 ± 306	566 ± 341	0.006
Chloride administered at 72 hours (mmol)	809 ± 425	809 ± 425	0.000

**Table 3:** Multivariate analysis

	Hospital mortality								
	24 Hour			48 Hour			72 Hour		
	Odd ratio	Confidence interval	P value	Odd ratio	Confidence interval	P value	Odd ratio	Confidence interval	P value
Variable									
APACHE 4 predicted mortality	14.352	8.84-23.31	0.000	14.6	8.88-24.31	0.000	11.74	7.51-18.35	0.000
Age	1.023	1.010-1.035	0.000	1.022	1.009-1.035	0.001	1.026	1.014-0.782	0.000
Total fluid administration	0.635	0.443-0.908	0.013	0.654	0.486-0.881	0.005	0.613	0.480-0.782	0.000
Chloride loading	1.004	1.001-1.007	0.013	1.004	1.001-1.006	0.003	1.005	1.003-1.007	0.000
pH	0.072	0.009-0.579	0.013	0.018	0.002-0.167	0.000	0.006	0.001-0.047	0.000
Chloride	1.004	0.977-1.032	0.763	1.020	0.990-1.051	0.188	1.003	0.998-1.052	0.071

## Conclusions

In conclusion, chloride administration in a critically ill population is an independent risk factor for mortality, even when corrected for total fluid administration, pH and chloride levels. Further studies the optimal resuscitation fluid with regard to chloride levels are needed.
